# Targeting NEK2 impairs oncogenesis and radioresistance via inhibiting the Wnt1/β-catenin signaling pathway in cervical cancer

**DOI:** 10.1186/s13046-020-01659-y

**Published:** 2020-09-10

**Authors:** Tie Xu, Yulan Zeng, Linli Shi, Qin Yang, Yeshan Chen, Gang Wu, Guiling Li, Shuangbing Xu

**Affiliations:** Cancer Center, Union Hospital, Tongji Medical College, Huazhong University of Science and Technology, Wuhan, 430022 China

**Keywords:** NEK2, Wnt1, β-Catenin, Radiosensitivity, Cervical cancer

## Abstract

**Background:**

NEK2, a serine/threonine kinase involved in mitosis, has been found to function in chromosome instability, tumor progression and metastasis, but its role in cervical cancer radioresistance remains unknown.

**Methods:**

We detected the protein levels of NEK2 in cervical carcinoma tissues and paired paracarcinoma tissues by immunohistochemistry. The roles of NEK2 in oncogenesis were examined using cell growth and colony formation assays, EdU assay, apoptosis assay as well as in vivo mouse model. γ-H2AX and Rad51 foci formation, neutral comet assay and clonogenic cell survival assay were applied to determine the radiosensitivity of cervical cancer cells. RNA-seq was performed to identify the downstream effector of NEK2. The gene expression levels were measured by Real-time PCR.

**Results:**

We report that NEK2 protein level is overexpressed and correlated with the tumor stage and lymph node metastasis in cervical cancer tissues. Furthermore, we provided evidence that depletion of NEK2 impairs oncogenesis and enhances radiosensitivity in cervical cancer. Using RNA sequencing, we identify Wnt1 as a key downstream effector of NEK2. Knockdown of NEK2 downregulates the mRNA and protein levels of Wnt1, thereby inhibiting the activation of the Wnt/β-catenin signaling pathway. More importantly, the observed consequences induced by NEK2 depletion in cervical cancer cells can be partially rescued by Wnt1 overexpression.

**Conclusions:**

Our results demonstrate that NEK2 activates the Wnt/β-catenin signaling pathway via Wnt1 to drive oncogenesis and radioresistance in cervical cancer, indicating that NEK2 may be a promising target for the radiosensitization of cervical cancer.

## Background

Cervical cancer remains the fourth most common female malignancy worldwide [1, 2]. Currently, surgery, radiotherapy, chemotherapy and immunotherapy are the main treatments for this type of cancer [3]. For locally advanced cervical cancer, concurrent radiochemotherapy have shown significant survival benefits [3]. However, cancer cell radioresistance is considered the leading cause of treatment failure. Thus, acquiring a deeper understanding of the mechanisms related to radioresistance and identifying novel therapeutic targets are crucial events for improving the survival of cervical cancer patients.

In humans, the Never in Mitosis A (NIMA)-related kinases family contains eleven serine/ threonine kinases which have been named as NEK1 to NEK11 [4]. Numerous studies have revealed that the NEK kinases participate in diverse cellular functions, including cell cycle control, centrosome organization, RNA splicing, inflammation and DNA damage response [5]. Of them, NEK1, NEK4, NEK5, NEK8, NEK10 and NEK11 have been linked to the DNA damage response [6–11]. For instance, NEK1 binds to ATR-ATRIP and promotes ATR signaling, whereas NEK11 controls the DNA damage checkpoint by directly phosphorylating and degrading Cdc25A [6, 7]. NEK5 silencing increases etoposide-induced DNA damage and impairs DNA repair, whereas NEK8 has been found to involve in replication fork stability through regulating Rad51 [10, 11]. All these data strongly support the notion that some of the NEK kinases are closely associated with DNA damage response and repair.

Never in Mitosis (NIMA)-related kinase 2 (NEK2) was initially identified as a key player in regulating mitotic processes, which include centrosome duplication and separation, microtubule organization and stabilization as well as spindle assembly checkpoint signaling [12]. Subsequently, more and more evidence has indicated that NEK2 is overproduced in various human cancers and participates in malignant transformation, including tumor progression and metastasis, drug resistance [13–15]. For instance, NEK2 phosphorylates p53 at Ser315 and reduces its stability, which functionally suppresses p53-mediated apoptosis to induce tumorigenesis [16]. It has also been documented that NEK2 depeltion impairs cancer cell drug resistance through inhibition of the PP1/AKT/NF-κB signaling pathway in multiple myeloma [17, 18]. Notably, a recent study reported that the mRNA expression level of NEK2 is significantly higher in invasive cervical cancer than in normal tissue [19], indicating that NEK2 may serve as a tumor-promoting protein in cervical cancer. Nevertheless, the exact roles and underlying mechanisms of NEK2 in cervical cancer progression and radioresistance has not yet been investigated.

Wnt signaling has been shown to play essential roles in the regulation of multiple biological processes, including cell proliferation, differentiation, migration and polarity, survival and self-renewal in stem cells [20–22]. Wnts act as positive regulators by inhibiting β-catenin degradation, stabilizing β-catenin, and causing β-catenin accumulation in the nucleus, ultimately controlling the expression of downstream target genes [23, 24]. Numurous studies have indicated that the deregulation of Wnt/β-catenin signaling is closely related to oncogenesis in several types of human cancers including breast cancer, hepatocellular carcinoma (HCC), ovarian cancer and colorectal cancer [25, 26]. Additionaly, Wnt/β-catenin signaling has also been revealed to mediate cancer radioresistance by participating in DNA damage repair [27, 28]. These data together support the idea that the activation of the Wnt/β-catenin signaling plays critical roles in oncogenesis and radioresistance.

In our study, we demonstrate that NEK2 protein levels are significantly upregulated and that elevated expression of NEK2 is correlated with the tumor stage and lymph node metastasis in cervical cancer. Furthermore, we identify Wnt1, a member of the Wnt family, as a key downstream effector of NEK2. Importantly, we show that NEK2 depletion impairs cervical cancer progression and radioresistance in a Wnt1-dependent manner, indicating that NEK2 may be a promising target for cervical cancer radiotherapy.

## Materials and methods

### Cell culture

Human cervical cancer cell lines HeLa and SiHa as well as HEK293T cells were purchased from the American Type Culture Collection and grown in DMEM medium supplemented with 10% fetal bovine serum and 100 μg/ml penicillin. All of the above cells were cultured at 37 °C in a humidified atmosphere containing 5% CO_2**.**_

### RNAi interference

The targeting siRNA sequences in this study were as follows: NEK2 siRNA#1, 5′-GGATCTGGCTAGTGTAATT-3′ and NEK2 siRNA#2, 5′-GCTAGAATATTAAACCATG-3′, which have been described previously [29]. HeLa and SiHa cells were transfected with indicated siRNAs (50 nM) using Lipofectamine RNAiMAX reagent (Invitrogen) according to the manufacturer‘s instructions. Subsequent experiments were performed 48 h post transfection.

### Establishment of stable NEK2-knockdown cervical cancer cell lines

Stable NEK2-knockdown cell lines were established as described previously [30, 31]. Briefly, HEK293T cells were transiently transfected with NEK2 shRNAs and pSPAX2 and pMD2G plasmids. Forty-eight hours post transfection, the lentivirus-containing supernatants were filtered and used to infect SiHa cells after mixing with 8 μg/ml polybrene to increase the infection efficiency. Stable cell lines were selected with 2 μg/ml puromycin and confirmed by Western blotting. The shRNA sequences used in our study were as follows:

Control shRNA: 5′-TTCTCCGAACGTGTCACGTTT-3′.

NEK2 shRNA-1: 5′-GGGATCTGAAACCAGCCAATG-3′.

NEK2 shRNA-2: 5′-GCATTAATGCCTCCATTTACA-3′.

### RNA sequencing

HeLa cells were transfected with control or NEK2-targeting siRNAs using Lipofectamine RNAiMAX for 48 h. Total RNA was isolated using TRIzol reagent (Invitrogen) according to the manufacturer’s instructions. Genes meeting the established threshold criteria of a false discovery rate (FDR) of < 5% and a fold change of > 2.0 were considered significantly differentially expressed.

### Reverse transcription and real-time PCR

Total cellular RNA was extracted using TRIzol reagent (Invitrogen). Reverse transcription was performed using a Prime RT reagent kit (Toyobo). Real-time PCR was performed using a SYBR® Premix Ex Taq™ Kit (Takara) according to the manufacturer’s instructions. The relative gene expression levels were calculated by the ΔCt method (the Ct of GAPDH minus the Ct of the target gene). Expression of GAPDH was used as the internal control. Primer sequences used for amplification were listed in Additional file 1: Table S1.

### Western blotting

Whole cell lysates were prepared in NETN buffer containing 20 mM Tris HCl (pH 8.0), 100 mM NaCl, 1 mM EDTA and 0.5% Nonidet P-40, separated on SDS-PAGE gels, and transferred to PVDF membranes. Western blotting was performed using the appropriate primary antibodies against NEK2 (1:200, sc55601, Santa Cruz Biotechnology), Wnt1 (1:500, ab15251, Abcam), Wnt4 (1:500, sc376279, Santa Cruz Biotechnology), β-catenin (1:1000, #8480, Cell Signaling Technology), Flag (1:1000, F1804, Sigma) and GAPDH (1:1000, #5174, Cell Signaling Technology) overnight at 4 °C. The PVDF membranes were then incubated with secondary antibodies and detected via enhanced chemiluminescence.

### Cell growth and colony formation assays

Transfected cells were plated in 6-well plates at a density of 1.0 × 10^4^ cells/ml, and the cell numbers in each well were evaluated every other day. Alternatively, cells were plated in 6-well plates at a certain density gradient and grown for two weeks. The colonies comprising more than 50 cells were counted.

### EdU assay

2 × 10^4^ cells in logarithmic growth phase were seeded in 96-well plates. The cells were harvested the next day and incubated with a 1/1000 dilution of EdU reagent for 0.5 h. The samples were washed with PBS and then incubated with 4% paraformaldehyde for 30 min. After being washed twice with PBS, the samples were permeabilized with 0.3% Triton X-100 in PBS and stained with reaction solution. Images were acquired via fluorescence microscopy.

### Apoptosis assay

After transfection with indicated siRNAs, the cells were collected and washed with cold PBS. Subsequently, the cells were analyzed using an Annexin V-PI Apoptosis Detection Kit I (BA1250, EnoGene, China) according to the manufacturer’s instructions. The apoptosis rates of HeLa and SiHa cells were analyzed by flow cytometry (Beckman, USA).

### Clonogenic cell survival assay

This assay was performed as described previously [32–34]. Cells transfected with the indicated siRNAs were seeded in triplicate into six-well plates and irradiated with indicated doses. After two weeks, the colonies comprising more than 50 cells were counted.

### Neutral comet assay

The neutral comet assay was performed using Trevigen comet assay kit according to the manufacturer’s instructions. Briefly, SiHa and HeLa cells transfected with indicated siRNAs were immobilized on the comet slide using low melting agarose, lysed overnight before being subjected to electrophoresis at 21 V for 30 min in a neutral unwinding buffer. Gels were then neutralized and stained with SYBR Gold (Invitrogen). Cells were photographed using a fluorescence microscope and the olive tail moment was analyzed by comet score software.

### Immunofluorescence staining

Cells were grown on coverslips and irradiated with 2 Gy. The cells were collected at 4 h after irradiation, fixed with 4% paraformaldehyde and permeabilized with 0.2% Triton X-100 for 5 min. Following three 5-min rinses with PBS, the coverslips were blocked with 5% bovine serum albumin. Finally, the samples were incubated with anti-γ-H2AX (1:500, ab26350, Abcam) or Rad51 antibody (1:500, ab63801, Abcam) overnight at 4 °C. The samples were then washed and incubated with Dylight549-conjugated goat anti-mouse IgG secondary antibody (1:200, A23310, Abbkine) for 1 h at room temperature. After counterstaining with DAPI, immunostained cells were examined with a fluorescence microscope.

### Immunohistochemical (IHC) staining

A cervical cancer tissue microarray, which contained 41 cervical carcinoma tissues and paired paracarcinoma tissues, was obtained from Shanghai Outdo Biotech (Shanghai, China). IHC analysis was performed as previously described [32–34]. Briefly, the tissue sections were deparaffinized, rehydrated, and blocked with goat serum. After incubation with the anti-NEK2 antibody (1:100, ab227958, Abcam) overnight at 4 °C, the sections were washed three times with PBS and incubated with an HRP-conjugated secondary antibody (1:300, K8002, Dako) for 30 min at room temperature. Following three 5-min rinses in PBS, the stained sections were reacted with 3,3′-diaminobenzidine for 10 min and then counterstained with 0.1% hematoxylin. The staining in tumor and normal tissues was scored, and the staining percentage was determined. The score calculated by multiplying the values assigned to the staining intensity and percentage was used to evaluate the expression of NEK2.

### In vivo xenografts mouse model

Animal experiment was approved by the Medical Ethics Committee of Tongji Medical College, Huazhong University of Science and Technology. This assay was performed as previously described [31, 32]. Briefly, 5-week-old female Balb/c mice were randomly grouped and injected subcutaneously with 2 × 10^7^ shControl, shNEK2#1 or shNEK2#1 SiHa cells. Tumors were measured and weighed every three or four days using calipers. The formula used to calculate tumor volumes was as follows: V = (L × W^2^)/2, where V = volume (mm^3^), L = length (mm), and W = width (mm).

### Statistical analyses

Each experiment in our study was performed independently with at least three replicates. All quantitative data are presented as the mean ± SD unless stated otherwise. Statistical data were analyzed using Statistical Program for Social Sciences (SPSS) 19.0 software (SPSS, Chicago, IL, USA). GraphPad Prism 6.0 (GraphPad Software, La Jolla, CA) was used to plot all graphs. Statistical differences between two groups were evaluated using a two-tailed Student’s t test. Pearson correlation analysis was applied to assess the correlation between NEK2 expression and clinicopathological parameters. Differences with *P* < 0.05 were considered statistically significant.

## Results

### NEK2 protein level is overexpressed and correlated with the tumor stage and lymph node metastasis in cervical cancer tissues

**Fig. 1:**
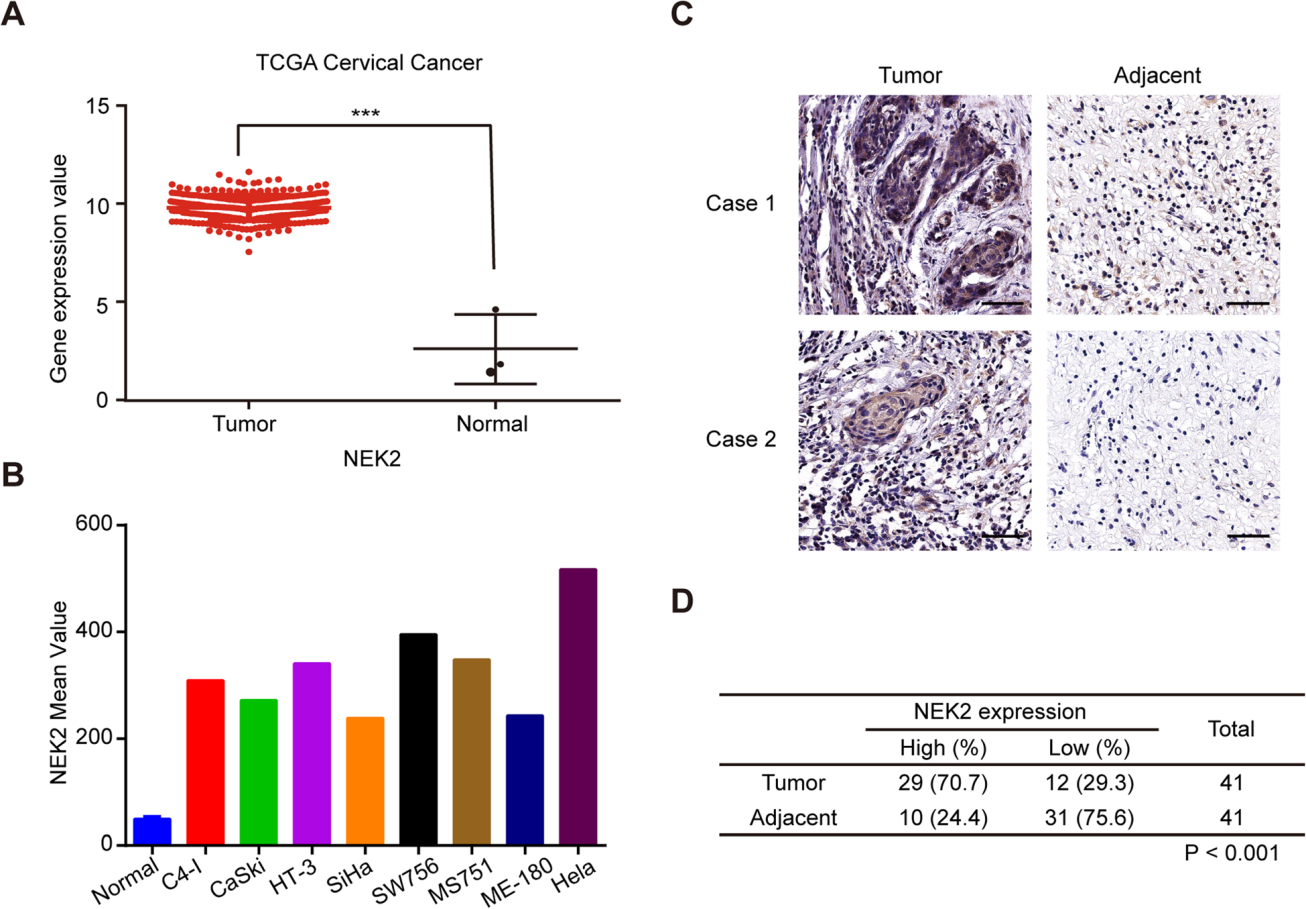
NEK2 is overexpressed in cervical cancer tissues. **a** The relative mRNA expression of NEK2 in cervical cancer tissues compared with normal tissues is shown in the TCGA database. **b** The mRNA expression analyses of NEK2 using GEO data set (GSE9750). Data was shown as the mean value of NEK2 from two independent data sets (GDS3233/204641 and GDS3233/211080). **c** Representative immunohistochemical staining images of NEK2 in cervical cancer tissues and paired adjacent tissues. Scale bar: 100 μm. **d** A summary of immunohistochemical staining of NEK2 expression in tissue microarrays constructed from cervical cancer and paired adjacent tissues

To investigate the clinical significance of NEK2 in cervical cancer, we first analyzed the transcriptomic profiles of cervical cancer and normal cervical tissues in The Cancer Genome Atlas (TCGA) database and found that NEK2 mRNA levels were significantly upregulated in cervical carcinoma tissue compared with normal tissue (Fig. 1a). In addition, our expression analyses using Gene Expression Omnibus (GEO) data set GSE9750 revealed that the mRNA levels of NEK2 were higher in human cervical cancer cell lines than that in normal cervix epithelial cells (Fig. 1b). To further validate whether NEK2 expression is indeed upregulated, we detected NEK2 protein level in 41 paraffin-embedded cervical cancer tissues and paired paracarcinoma tissues by immunohistochemistry. As shown in Fig. 1c and d, NEK2 was primarily localized in the nucleus and was overexpressed in cervical cancer tissues. NEK2 positivity was dramatically higher in cervical cancer tissues (70.7%) than in the adjacent paracarcinoma tissues (24.4%) (*P* < 0.001). Notably, a significant correlation was found between NEK2 expression and the tumor stage as well as lymph node metastasis in 123 paraffin-embedded cervical cancer tissues (*P* < 0.05) (Additional file 2: Table S2). Together, these results indicate that NEK2 upregulation may contribute to oncogenesis in cervical cancer.

### Loss of NEK2 suppresses oncogenesis in cervical cancer in vitro and in vivo

**Fig. 2:**
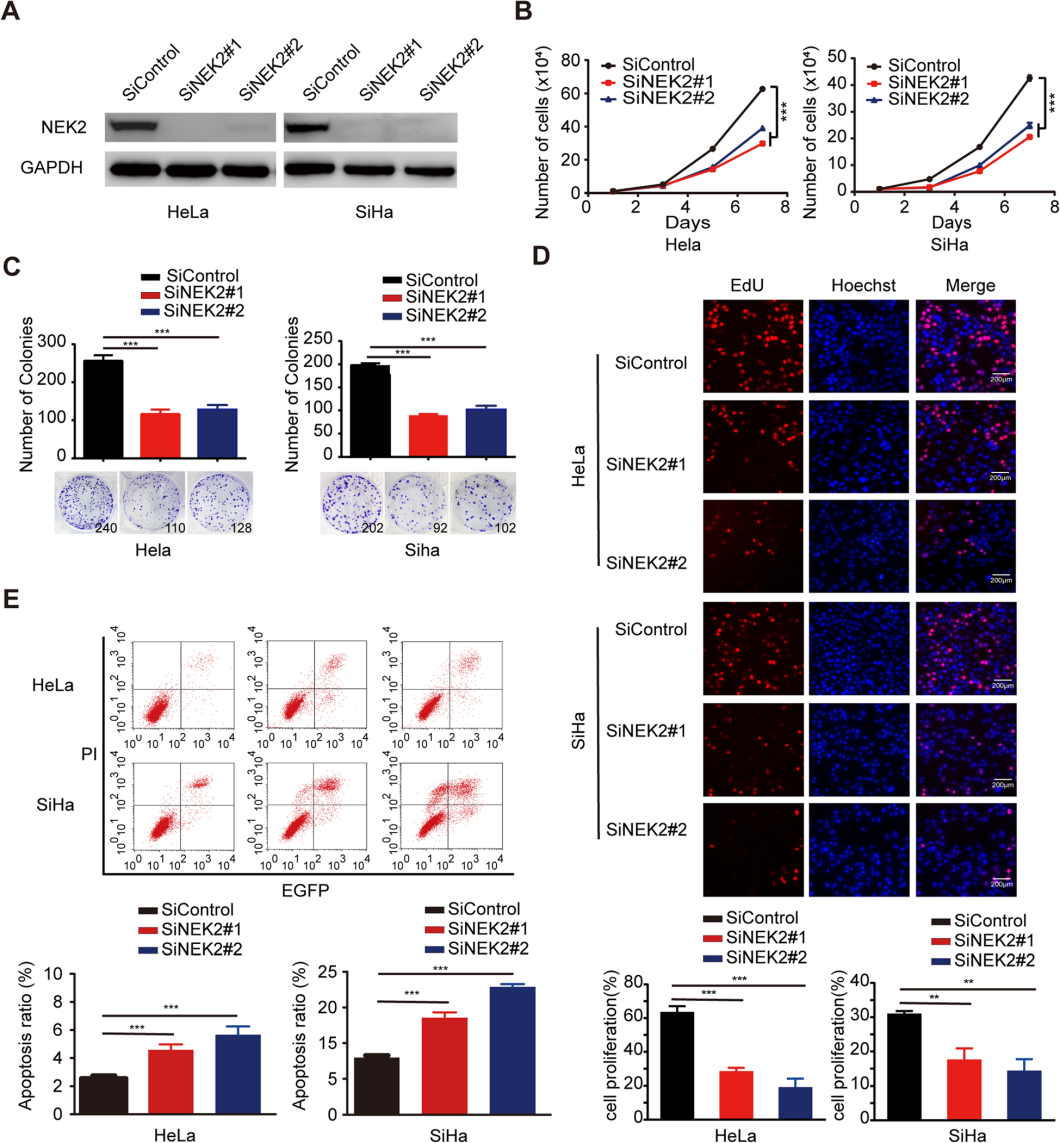
NEK2 silencing suppresses the growth and proliferation and induces the apoptosis of cervical cancer cells in vitro*.* **a** HeLa and SiHa cells were transfected with indicated siRNAs for 48 h, and samples were harvested and analyzed by Western blotting with the indicated antibodies (*n* = 3). **b** NEK2 silencing inhibited the growth of HeLa and SiHa cells (n = 3). **c** Colony formation ability was significantly reduced in NEK2-depleted cells. *** *P* < 0.001 (n = 3). **d** Knockdown of NEK2 reduced the proliferation of HeLa and SiHa cells, as determined by an EdU cell proliferation assay. ** *P* < 0.01, *** P < 0.001 (n = 3). Scale bar: 200 μm. **e** HeLa and SiHa cells transfected with indicated siRNAs for 48 h were subjected to annexin V-EGFP/propidium iodide staining, and analyzed by flow cytometry. *** P < 0.001 (n = 3)

Given that NEK2 is overexpressed in cervical cancer tissues, we speculated that it may act as an oncogenic protein in cervical cancer. To test this hypothesis, we first downregulated the expression level of NEK2 in two different cervical cancer cell lines (HeLa and SiHa) by siRNA transfection. As shown in Fig. 2a, NEK2 protein expression was successfully knocked down. As expected, NEK2 silencing suppressed the growth of cervical cancer cells compared with control cells (Fig. 2b and Additional file 3: Figure S1). The colony formation ability of cells was also impaired when NEK2 was knocked down (Fig. 2c). Consistent with this notion, the 5-ethynyl-2′-deoxyuridine (EdU) staining assay showed that the proliferation of cervical cancer cells was inhibited when NEK2 was depleted (Fig. 2d). We then determined the ratio of apoptotic cells, as measured by Annexin V/PI staining and flow cytometry. As shown in Fig. 2e, downregulation of NEK2 resulted in increased proportions of apoptotic cells. These findings demonstrate that NEK2 promotes the growth and proliferation and inhibits the apoptosis of cervical cancer cells.

**Fig. 3:**
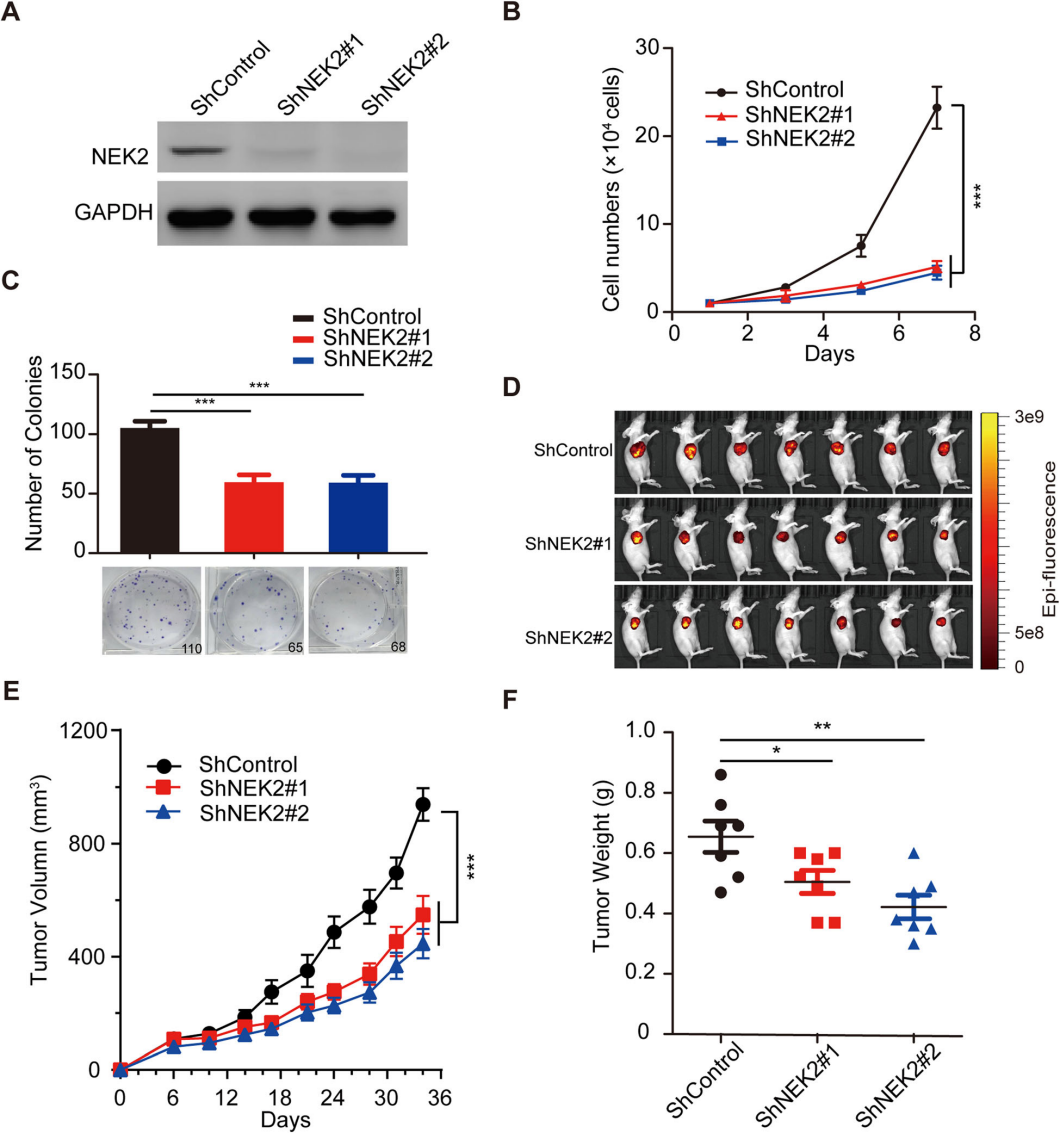
Downregulation of NEK2 inhibited the oncogenic behavior of cervical cancer cells in vitro and in vivo*.* **a** Western blotting analyses showed that NEK2 was efficiently knocked down in SiHa cells by ShRNA transfection. **b** Stable knockdown of NEK2 suppressed SiHa cell growth in vitro. *** *P* < 0.001 (*n* = 3). **c** Colony formation ability was reduced in the ShNEK2 groups compared with the ShControl group. *** P < 0.001 (*n* = 3). **d** Representative images of the xenograft tumors in three groups (*n* = 7 mice/group). **e** The growth curves of the xenograft tumors in three groups were presented. Tumor volumes were calculated every three or four days. Data was shown as the mean tumor volume ± SEM (n = 7 mice/group). *** *P* < 0.001. **f** The tumor weights in three groups were presented. * *P* < 0.05, ** *P* < 0.01

To further confirm the role of NEK2 in promoting oncogenesis in cervical cancer, we constructed stable NEK2-knockdown SiHa cells using NEK2 shRNAs (Fig. 3a) and found that shRNAs-mediated knockdown of NEK2 significantly inhibits cervical cancer cell growth and proliferation (Fig. 3b and c), which is consistent with above findings. To investigate whether NEK2 drives oncogenesis in vivo, we subcutaneously implanted shcontrol or shNEK2 cells into T-cell-deficient athymic nude mice. As shown in Fig. 3d-f, the ShNEK2 groups displayed a reduction in the tumor size and weight compared to ShControl group. Taking into account the above results, we conclude that loss of NEK2 impairs oncogenesis in cervical cancer in vitro and in vivo.

### NEK2 silencing enhances the radiosensitivity of cervical cancer cells by increasing radiation-induced DNA damage and inhibiting DNA repair

**Fig. 4:**
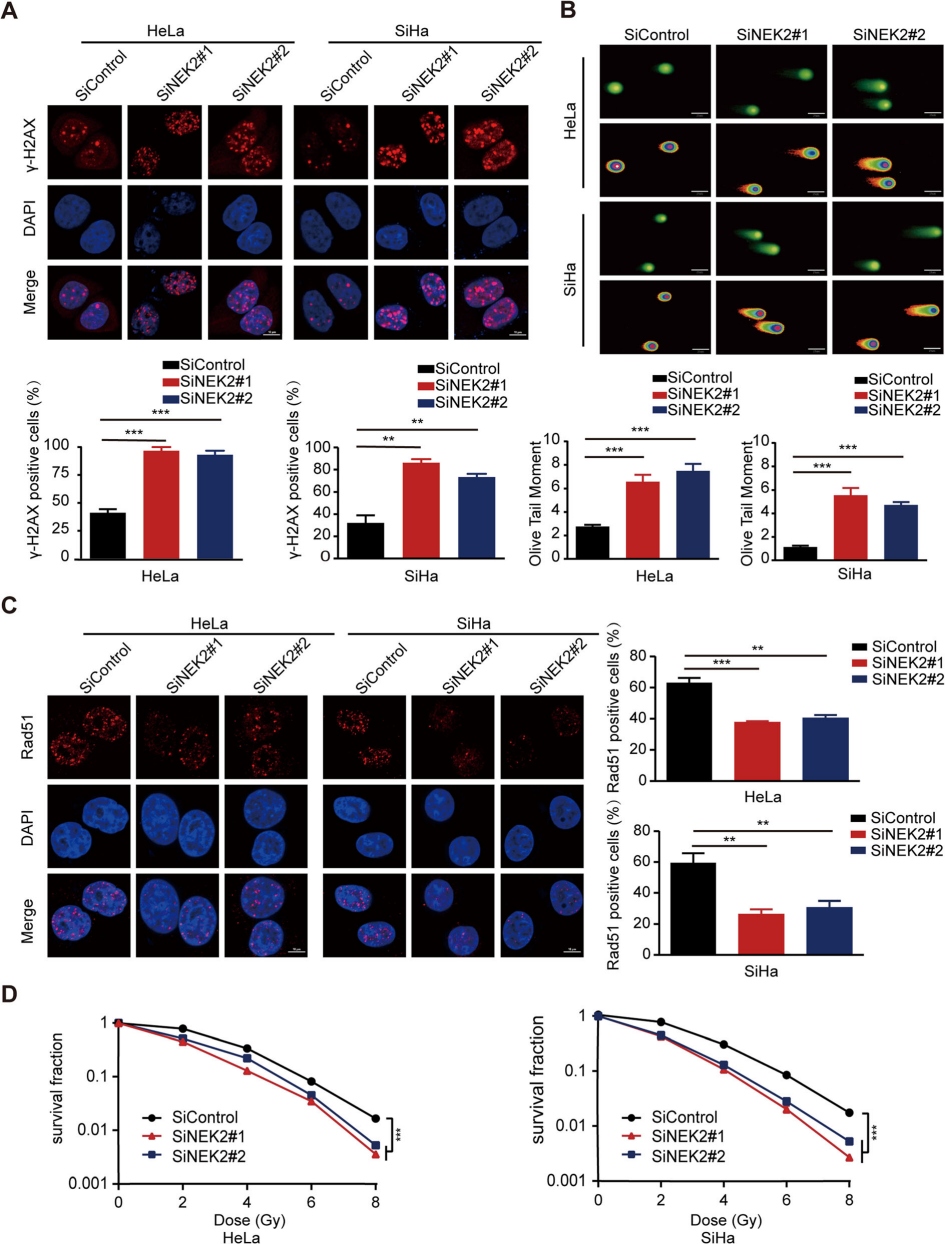
Loss of NEK2 enhances the radiosensitivity of cervical cancer cells. **a** Upper panel: HeLa and SiHa cells transfected with indicated siRNAs were exposed to radiation and harvested 4 h later. Immunostaining was performed to detect γ-H2AX foci formation. Lower panel: Quantification result of γ-H2AX foci was shown. Cell containing more than ten γ-H2AX foci was considered γ-H2AX-positive cell. ** *P* < 0.01, *** *P* < 0.001 (*n* = 3). Scale bar: 10 μm. **b** Upper panel: Representative pictures of neutral comet assay performed 4 h after IR exposure of NEK2-depleted or control cells. Lower panel: The olive tail moment was quantified and graphed. *** *P* < 0.001 (*n* = 3). Scale bar: 25 μm. **c** Left panel: HeLa and SiHa cells transfected with indicated siRNAs were exposed to radiation and harvested 4 h later. Immunostaining was performed to determine Rad51 foci formation. Right panel: Quantification result of Rad51 foci was shown. ** *P* < 0.01, *** *P* < 0.001 (*n* = 3). Scale bar: 10 μm. **d** Radiation sensitivity of HeLa and SiHa cells lacking NEK2. HeLa and SiHa cells transfected with indicated siRNAs were irradiated at indicated doses. The colonies were counted two weeks later. Data were shown as the mean ± SD. *** P < 0.001 (n = 3)

After investigating the role of NEK2 under physiological conditions, we also sought to determine whether NEK2 participates in cancer radioresistance under radiation-induced DNA damage. To this end, we first performed γ-H2AX foci formation assay since γ-H2AX foci is considered a key marker of DNA damage response [35]. As shown in Fig. 4a, the percentage of γ-H2AX foci-positive cells was dramatically increased in NEK2-depleted cervical cancer cells after irradiation exposure. In addition, the comet tail moment, which also reflects DNA damage, were significantly increased in irradiated NEK2 deficiency cells (Fig. 4b). These results clearly indicate that NEK2 knockdown accelerates DNA damage. It has been well established that Rad51 is an essential modulator of homologous recombination (HR) related to DNA repair [36]. Our results demonstrated that DNA damage-induced Rad51foci formation was significantly decreased upon NEK2 silencing (Fig. 4c), indicating that loss of NEK2 impairs DNA repair. In line with these ideas, NEK2 knockdown enhanced cellular sensitivity to radiation compared with that of control cells (Fig. 4d). Collectively, our results support the notion that NEK2 promotes the radioresistance of cervical cancer cells.

### NEK2 controls the expression of Wnt1 and activates the Wnt1/β-catenin signaling pathway

**Fig. 5:**
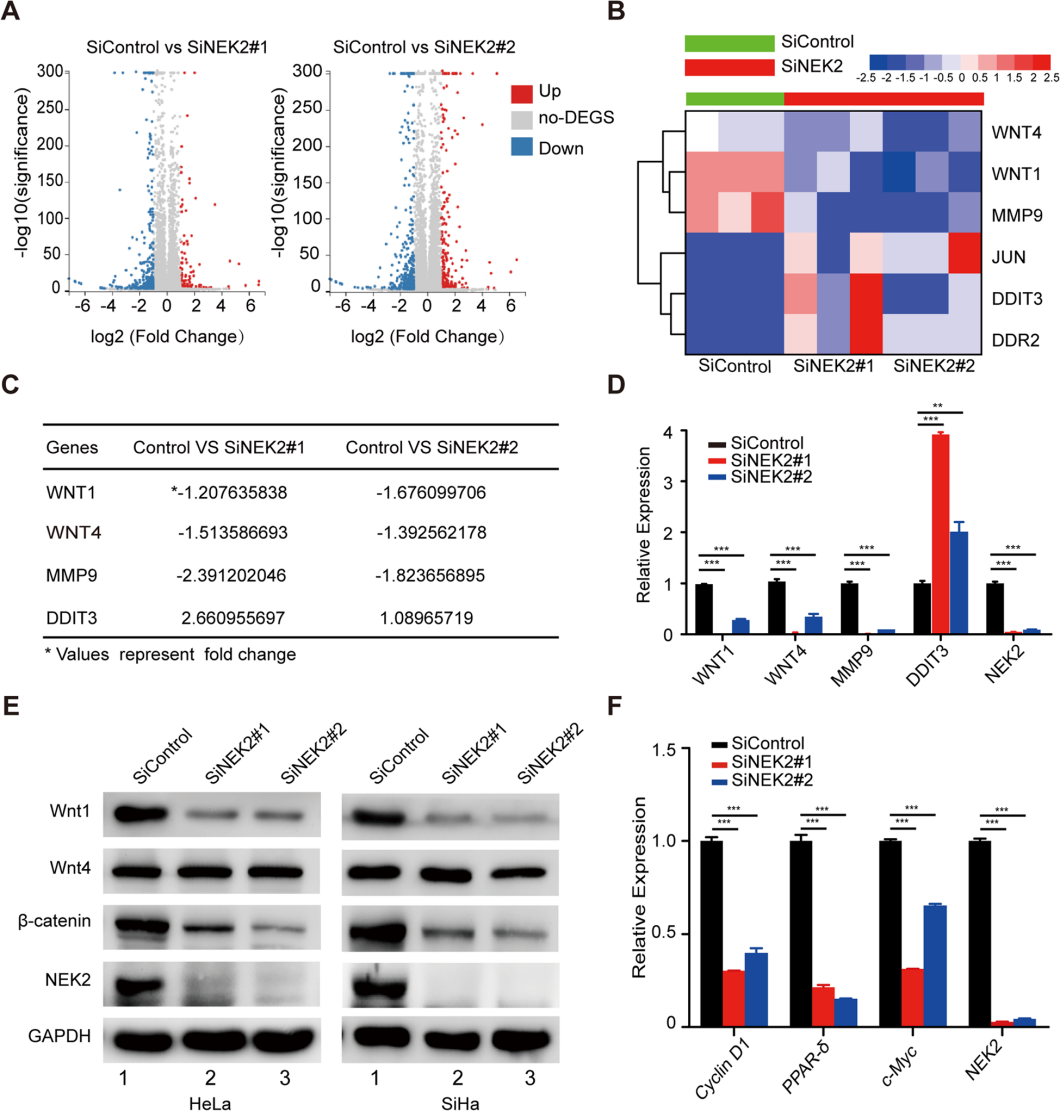
NEK2 silencing downreglates the expression of Wnt1 and attenuates the Wnt1/β-catenin signaling pathway. **a** Volcano plot of differentially expressed genes resulting from NEK2-depleted and control HeLa cells: the X-axis indicates the log2 transformed expression values, and the Y-axis represents the significance value after -log10 transformation. Red indicates upregulated differentially expressed genes, blue indicates downregulated differentially expressed genes, and gray indicates non-differentially expressed genes. **b** Heat map produced from RNA-seq data. HeLa cells were transfected with the indicated siRNAs for 48 h, and total mRNA was subjected to RNA-seq analysis. **c** List of several differentially expressed genes related to cell proliferation (Fold change > 2). **d** HeLa cells were transfected with the indicated siRNAs. After 48 h, the cells were harvested, and the levels of indicated mRNAs were measured by quantitative real-time PCR. ** P < 0.01, *** P < 0.001 (n = 3). **e** Knockdown of NEK2 led to decreased protein levels of Wnt1 and β-catenin in HeLa and SiHa cells (n = 3). **f** HeLa cells transfected with the indicated siRNAs for 48 h were collected, and the levels of indicated mRNAs were determined by quantitative real-time PCR. *** P < 0.001 (n = 3)

To explore the underlying mechanisms that contribute to the effects of NEK2 in cervical cancer, we performed RNA sequencing (RNA-seq) to compare the genomic expression profiles of HeLa cells transfected with control or NEK2-targeting siRNAs. As shown in Fig. 5a and b, there are many differentially expressed genes with a fold change of greater than two in NEK2 depleted cells. Among these genes, we selected four candidate genes (WNT1, WNT4, MMP9 and DDIT3) that have been reported to be involved in oncogenesis to verify (Fig. 5c). Our real-time PCR results showed that WNT1,WNT4 and MMP9 were significantly downregulated while DDIT3 was upregulated after NEK2 depletion (Fig. 5d), which is consistent with the RNA-seq data. Notably, the protein level of Wnt1 but not Wnt4 was also remarkably decreased when NEK2 was knocked down (Fig. 5e), indicating that NEK2 controls the expression of Wnt1 at the mRNA and protein levels. It has been shown that Wnt1 is the activator of Wnt/β-catenin signaling pathway [37]. To elucidate whether NEK2 is related to this classical oncogenic pathway, we examined the expression of β-catenin and found that NEK2 silencing led to significant reduction in β-catenin at the protein level in two different cervical cancer cell lines (Fig. 5e). Interestingly, bioinformatics analysis with the TCGA database showed that there was a positive correlation between NEK2 and β-catenin mRNA expression in cervical cancer tissues (Additional file 4: Figure S2), further suggesting that NEK2 may be a critical regulator of Wnt/β-catenin signaling in cervical cancer. In addition, we also demonstrated that Cyclin D1, PPAR-δ and c-Myc, which represent downstream target genes of Wnt/β-catenin pathway, were downregulated in NEK2 deficiency cells (Fig. 5f). These results indicate that NEK2 modulates the expression of Wnt1 to activate the Wnt1/β-catenin signaling pathway.

### The decreased proliferation and enhanced radiosensitivity of cervical cancer cells caused by NEK2 silencing are mainly dependent on Wnt1

**Fig. 6:**
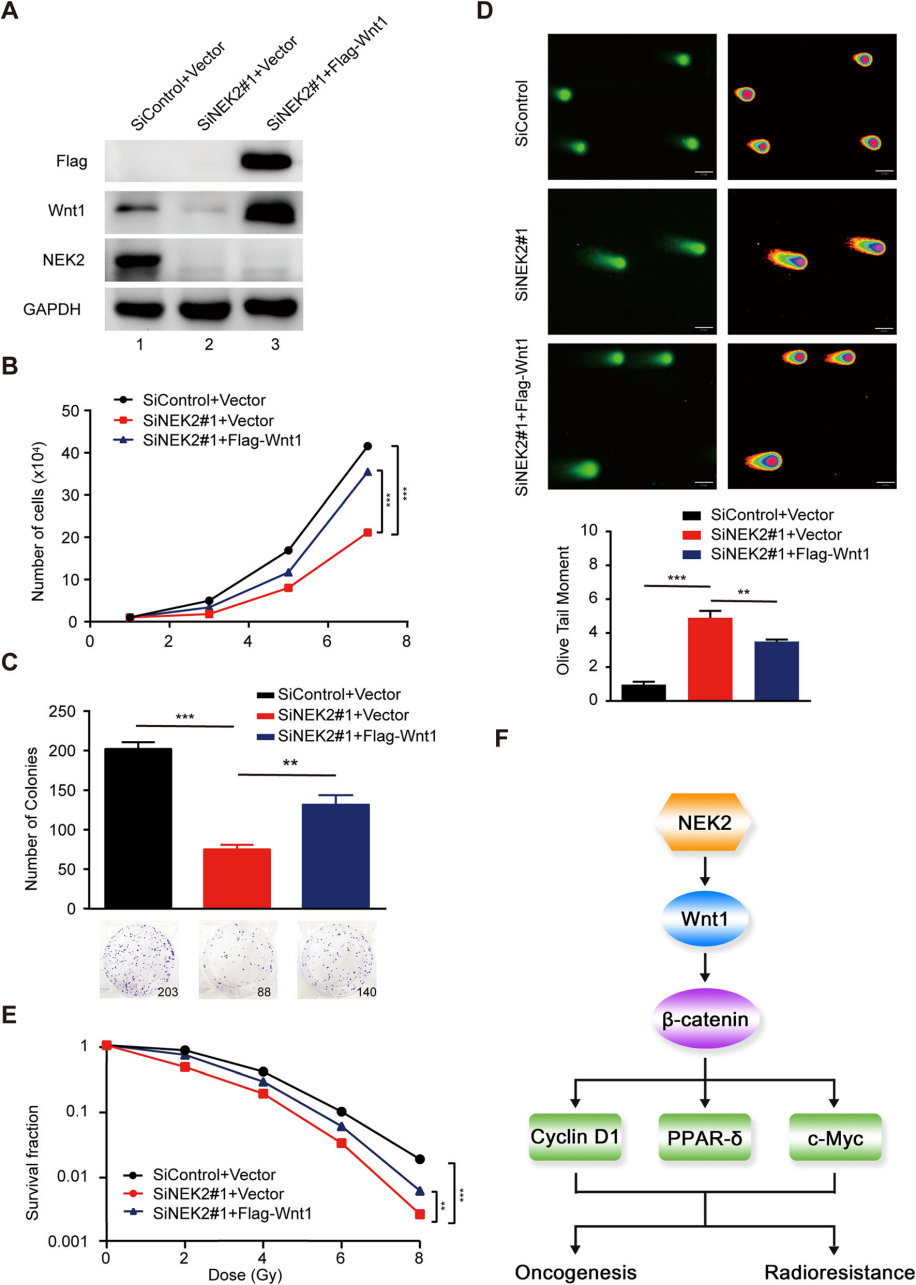
Wnt1 overexpression partially rescues the impaired proliferation and radioresistance in cervical cancer cells. **a** NEK2 knockdown SiHa cells were reconstituted with empty vector or Flag-tagged Wnt1 vector (n = 3). **b** SiHa cells transfected with the indicated siRNAs and plasmids were seeded, and cell numbers were calculated every other day. *** P < 0.001 (n = 3). **c** Upper panel: SiHa cells transfected with the indicated siRNAs and plasmids were seeded and grown for two weeks. The number of colonies was counted. Lower panel: Colony formation was quantified. ** P < 0.01, *** P < 0.001 (n = 3). **d** Neutral comet assay was performed after exposure to irradation. Upper panel: Representative images showing olive tail moment. Scale bar, 50 μm. Lower panel: the olive tail moment was quantified and graphed. ** P < 0.01, *** P < 0.001 (n = 3). **e** SiHa cells were irradiated with the indicated doses after transfection with the indicated siRNAs and plasmid. Colony survival rates were determined two weeks later. ** P < 0.01, *** P < 0.001 (n = 3). **f** A proposed model illustrating the mechanism by which NEK2 promotes cervical cancer oncogenesis and radioresistance. NEK2 upregulates Wnt1 and activates the Wnt/β-catenin signaling pathway, thereby inducing the expression of β-catenin downstream target genes and ultimately promoting oncogenesis and radioresistance in cervical cancer

To investigate whether Wnt1 is indeed required for the observed phenotypes caused by NEK2 silencing in cervical cancer cells, we transfected exogenously expressed Wnt1 into NEK2-depleted cells (Fig. 6a). As shown in Fig. 6b and c, the defects in cell growth and proliferation induced by NEK2 knockdown were partially rescued by overexpression of Wnt1. Similarly, restoration of Wnt1 expression partially reversed the increased olive tail moment induced by NEK2 silencing in response to DNA damage (Fig. 6d). Additionally, the enhanced radiosensitivity caused by NEK2 depletion was also partially restored by transfection with a plasmid encoding Wnt1 (Fig. 6e). Accordingly, these results reveal that NEK2 exerts its biological functions mainly via regulating Wnt1 in cervical cancer.

## Discussion

In this study, we report that NEK2 protein is overexpressed and correlated with the tumor stage and lymph node metastasis in cervical cancer tissues. Moreover, we for the first time show that NEK2 upregulates Wnt1 to activate Wnt/β-catenin signaling pathway, thereby promoting oncogenesis and radioresistance in cervical cancer. Our findings indicate that targeting NEK2/Wnt1/β-catenin pathway may be a potential radiosensitization strategy in cervical cancer.

NEK2 was initially identified as a serine/threonine kinase with roles in cell cycle and mitosis regulation [38]. Subsequently, aberrant expression of NEK2 has been observed in several types of human cancers [13]. However, few studies have examined the effects of NEK2 on tumor aggressiveness and radiotherapy resistance in cervical cancer. Our clinical data demonstrated that NEK2 is overexpressed in cervical cancer and associated with the tumor stage and lymph node metastasis, indicating that NEK2 may act as an oncoprotein involved in cervical cancer tumorigenesis. Subsequent functional studies confirmed this notion that loss of NEK2 suppresses tumorigenesis in vitro and in vivo, indicating that NEK2 plays oncogenic role in cervical cancer. In addition, we also revealed the role of NEK2 in driving radioresistance. NEK2 knockdown amplifies DNA damage signal and impedes DNA repair, ultimately leading to enhanced radiosensitivity in cervical cancer. Although several other NEK kinases have been implicated in the DNA damage response, our study reveal for the first time that NEK2 has a previously unknown role in promoting cervical cancer radioresistance, further supporting that NEK kinases act as critical regulators in the DNA damage repair process.

Wnt/β-catenin pathway is a highly conserved and tightly regulated signaling that controls diverse physiological and pathological processes including carcinogenesis [37]. This pathway has been shown to contribute to cervical cancer pathology in various stages, including tumor initiation, progression, invasion, and therapeutic resistance [39, 40]. As a member of the Wnt family, Wnt1 has been shown to involve in tumor progression, adaptive immune resistance and bone remodeling [41–43]. In our study, we identified Wnt1 as a key downstream effector of NEK2 in cervical cancer. NEK2 silencing led to a significant reduction of Wnt1 at both the transcriptional and translational levels, which in turn attenuate the Wnt/β-catenin signaling pathway. Importantly, our rescue results showed that the biological effects caused by NEK2 knockdown are mainly dependent on Wnt1 in cervical cancer cells. Accordingly, our work uncovered that NEK2 is a novel positive modulator of Wnt1 and provided new insights into the molecular mechanisms by which NEK2 participates in oncogenesis and radioresistance in cervical cancer.

## Conclusions

In summary, we reveal that NEK2 induces the expression of Wnt1 to activate the Wnt/β-catenin signaling pathway, leading to oncogenesis and radioresistance in cervical cancer, as proposed in Fig. 6f. Given that NEK2 is overexpressed and promotes radioresistance in cervical cancer, targeting NEK2 may be a desirable therapeutic strategy for cervical cancer radiotherapy.

## Supplementary information

**Additional file 1: Table S1.** Sequences of primers used for Real-time quantitative PCR.

**Additional file 2: Table S2.** Correlation between the clinicopathologic variables and expression of NEK2 in 123 paraffin-embedded cervical cancer tissues.

**Additional file 3: Figure S1.** Cell growth was suppressed in NEK2 deficiency cells. *** *P* < 0.001 (*n* = 3).

**Additional file 4: Figure S2.** Scatterplots of NEK2 vs β-catenin mRNA expression in cervical cancer samples available from the TCGA database (*n* = 309). The Pearson correlation coefficient (r) and *P* value are shown.

## Data Availability

The analyzed datasets generated during the current study are available from the corresponding author on reasonable request.

## Abbreviations

NEK2: Never in Mitosis (NIMA)-related kinase 2
HCC: Hepatocellular carcinoma
DMEM: Dulbecco’s modified Eagle medium
FDR: False discovery rate
IHC: Immunohistochemical
TCGA: The Cancer Genome Atlas
HR: Homologous recombination
RNA-seq: RNA sequencing
