# Supplementary material for: Targeting NEK2 impairs oncogenesis and radioresistance via inhibiting the Wnt1/β-catenin signaling pathway in cervical cancer
Source: J Exp Clin Cancer Res. 2020 Sep 10;39:183. doi: 10.1186/s13046-020-01659-y (PMC7488040; doi:10.1186/s13046-020-01659-y)
Supplement: Supplementary file 1 — Additional file 1: Table S1. Sequences of primers used for Real-time quantitative PCR. [file 13046_2020_1659_MOESM1_ESM.docx]

**Additional file 1: Table S1.** Sequences of primers used for Real-time quantitative PCR

| Genes | Sequences (5'--3') | Size (bp) |
| --- | --- | --- |
| NEK2 | F: TCCCCACTGAAATGAACTTTCT  R: CAGCTTGCTAAAGGAACGGA | 102 |
| WNT1 | F: TGGCTGGGTTTCTGCTACG  R: CCCGGATT TTGGCGTATC | 207 |
| WNT4 | F: GAGGAGACGTGCGAGAAACTCAA  R: ATCCTGACCACTGGAAGCCCTGT | 346 |
| MMP9 | F: GCACCACCACAACATCAC  R:ACCACAACTCGTCATCGTC | 284 |
| DDIT3 | F: GCGACAGAGCCAGAATAACAGC  R:TTCTGCTTTCAGGTGTGGTGGT | 90 |
| Cyclin D1 | F: GATGCCAACCTCCTCAACGAC  R:CTCCTCGCACTTCTGTTCCTC | 171 |
| PPARδ | F: CACATCTACAATGCCTACCT  R:CTTCTCTGCCTGCCACAATGTCT | 132 |
| c-Myc | F: CAGCGACTCTGAGGAGGAAC  R:TGTGAGGAGGTTTGCTGTGG | 131 |
| GAPDH | F: GCTGAGAACGGGAAGCTTGT  R: GCCAGGGGTGCTAAGCAG | 299 |

*^a^* F, forward primer; R, reverse primer.
