# Supplementary material for: Targeting NEK2 impairs oncogenesis and radioresistance via inhibiting the Wnt1/β-catenin signaling pathway in cervical cancer
Source: J Exp Clin Cancer Res. 2020 Sep 10;39:183. doi: 10.1186/s13046-020-01659-y (PMC7488040; doi:10.1186/s13046-020-01659-y)
Supplement: Supplementary file 2 — Additional file 2: Table S2. Correlation between the clinicopathologic variables and expression of NEK2 in 123 paraffin-embedded cervical cancer tissues. [file 13046_2020_1659_MOESM2_ESM.docx]

| Features | NEK2 expression | |  |
| --- | --- | --- | --- |
|  | High group | Low group | P value |
| **Age (years)** |  |  |  |
| ≤65 | 65 | 51 | 0.424 |
| >65 | 5 | 2 |  |
| **Tumor stage** |  |  |  |
| Ⅰ | 4 | 2 | 0.197 |
| Ⅱ | 8 | 13 |  |
| Ⅲ | 46 | 34 |  |
| **T** |  |  |  |
| T1 | 44 | 24 | **0.046** |
| T2 | 15 | 14 |  |
| T3 | 9 | 14 |  |
| T4 | 2 | 1 |  |
| **N** |  |  |  |
| N0 | 61 | 38 | **0.032** |
| N1 | 9 | 15 |  |
| **M** |  |  |  |
| M0 | 70 | 52 | 0.252 |
| M1 | 0 | 1 |  |
| **Relapse** | |  |  |
| yes | 28 | 14 | 0.116 |
| no | 42 | 39 |  |

**Additional file 2 Table S2.** Correlation between the clinicopathologic variables and expression of NEK2 in 123 paraffin-embedded cervical cancer tissues
