# Supplementary figures and images for: Targeting NEK2 impairs oncogenesis and radioresistance via inhibiting the Wnt1/β-catenin signaling pathway in cervical cancer
Source: J Exp Clin Cancer Res. 2020 Sep 10;39:183. doi: 10.1186/s13046-020-01659-y (PMC7488040; doi:10.1186/s13046-020-01659-y)

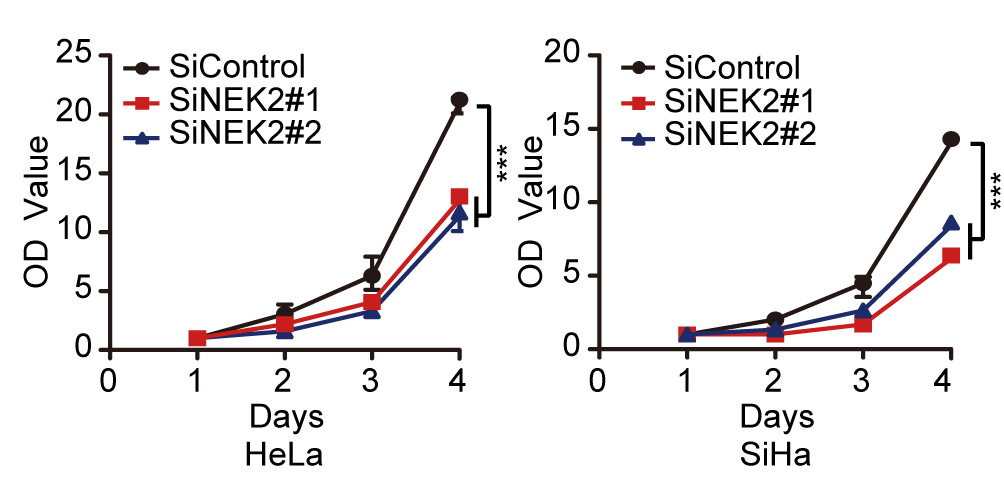

Supplement: Supplementary file 3 — Additional file 3: Figure S1. Cell growth was suppressed in NEK2 deficiency cells. *** P < 0.001 (n = 3). [file 13046_2020_1659_MOESM3_ESM.tif]

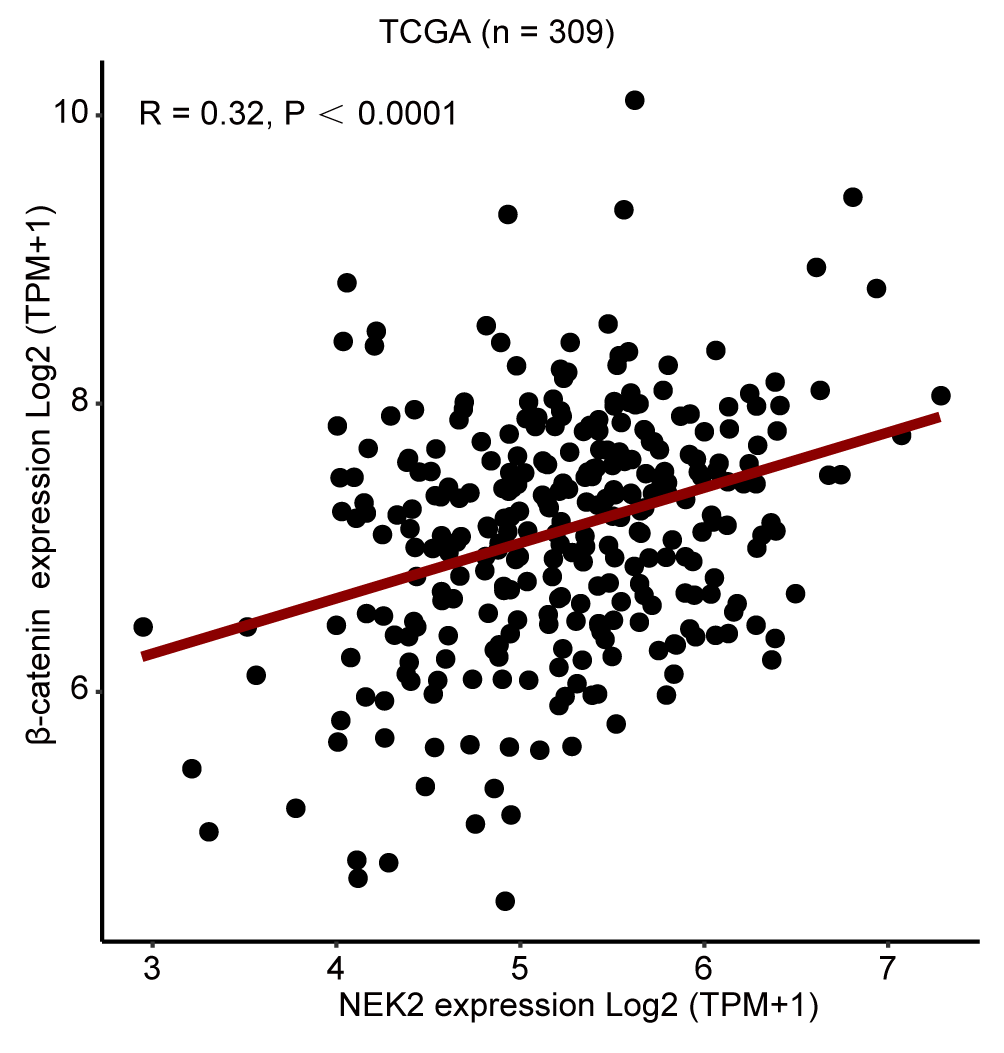

Supplement: Supplementary file 4 — Additional file 4: Figure S2. Scatterplots of NEK2 vs β-catenin mRNA expression in cervical cancer samples available from the TCGA database (n = 309). The Pearson correlation coefficient (r) and P value are shown. [file 13046_2020_1659_MOESM4_ESM.tif]
